# SeqGene: a comprehensive software solution for mining exome- and transcriptome- sequencing data

**DOI:** 10.1186/1471-2105-12-267

**Published:** 2011-06-29

**Authors:** Xutao Deng

**Affiliations:** 1 Bioinformatics Core Facility, Department of Molecular Medicine, Beckman Research Institute, City of Hope Medical Center, Duarte, CA 91010, USA

## Abstract

**Background:**

The popularity of massively parallel exome and transcriptome sequencing projects demands new data mining tools with a comprehensive set of features to support a wide range of analysis tasks.

**Results:**

SeqGene, a new data mining tool, supports mutation detection and annotation, dbSNP and 1000 Genome data integration, RNA-Seq expression quantification, mutation and coverage visualization, allele specific expression (ASE), differentially expressed genes (DEGs) identification, copy number variation (CNV) analysis, and gene expression quantitative trait loci (eQTLs) detection. We also developed novel methods for testing the association between SNP and expression and identifying genotype-controlled DEGs. We showed that the results generated from SeqGene compares favourably to other existing methods in our case studies.

**Conclusion:**

SeqGene is designed as a general-purpose software package. It supports both paired-end reads and single reads generated on most sequencing platforms; it runs on all major types of computers; it supports arbitrary genome assemblies for arbitrary organisms; and it scales well to support both large and small scale sequencing projects. The software homepage is http://seqgene.sourceforge.net.

## Background

Massively parallel sequencing of exome and transcriptome has been widely adopted to effectively interrogate the key protein-coding and non-coding RNA regions. Exome sequencing (exome-Seq) technology has been especially effective for identifying single-nucleotide polymorphisms (SNPs) and small insertions/deletions (indels) that may cause diseases and other phenotypes. To name a few examples, Ng et al. [1] have found that the mutations of DHODH gene causes Miller syndrome, a Mendelian disorder, by sequencing four affected exomes in three independent kindreds. Yi et al., [2] sequenced 50 exomes of ethnic Tibetans and successfully identified a mutation at EPAS1 gene that is associated with adaptation to high altitude. For quantitative RNA abundance measurement, RNA sequencing (RNA-Seq) compares favourably to other methods, such as gene expression microarrays. The benefits of using RNA-Seq include high resolution, high dynamic range of expression, low background noise, and the ability to identify allele specific expression and different isoforms [3-6].

However, exome-Seq and RNA-Seq face several bioinformatic challenges, including the development of efficient methods to perform basecalling, assembly, alignment and post-alignment on large amounts of data. There listed more than 350 software tools on http://seqanswers.com[7] including more than 100 for alignment, more than 50 for sequence assembly, more than 10 for basecalling, and many others for performing various post-alignment analysis tasks. However, most of the post-alignment open source software tools have very limited features and support only one or few analysis tasks. To name a few that relates to our work, ERANGE [8] is a tool for RNA-Seq expression normalization and quantification; SAMtools [9] is mainly developed for alignment format conversion and SNP/indel calling; GAMES [10] supports exome-Seq mutation discovery and functional annotation; DEGseq [11] supports finding differentially expressed genes from RNA-Seq data. Using a combination of software tools for various analytical purposes presents a challenge to investigators because the tools often require different hardware specification, operating systems and incompatible data formats. Therefore, there is an urgent need for new exome-Seq and RNA-Seq software tools with a relatively rich feature set that is accessible to investigators with limited or no programming skills to facilitate their multi-analysis requests. We therefore developed SeqGene, an open-source software tool which integrates mutation identification, annotation, genotyping, expression quantification, copy number variation (CNV), expression quantitative trait loci (eQTLs) detection, allele specific expression (ASE), differentially expressed genes (DEGs) identification, and pathway analysis workflows in a single package. SeqGene also implements several novel functions that we proposed, such as a new method for SNP identification and filtering, a new SNP-expression association test based on KEGG-pathways, and a new method for genotype-controlled differentially expressed genes (GCDEG) identification.

## Methods

**Figure 1:**
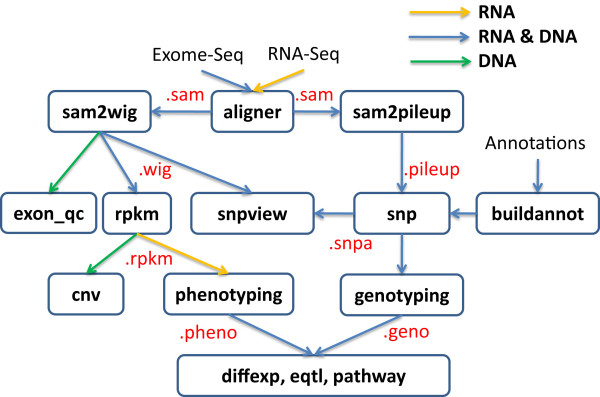
**The structure of SeqGene**. The diagram shows the relationship between the functions implemented in SeqGene. The rectangles represent the functions; the arrows represent input and output, and the file formats are in the red font.

The major components of SeqGene are illustrated in Figure 1, where the functions were represented in the rectangles, the relationship between them and the corresponding input and out files were shown by arrows, and the file formats are in the red font. Below we explain each major function in more detail.

### Mutation detection and annotation

Detecting genomic variants (such as SNPs, indels and structural variants) via whole-genome sequencing, RNA-Seq and exome-Seq is an essential approach to understanding the association of genotypic difference to phenotypic consequences with the eventual goal of personalized genomics for medical purposes [1,12-15]. Among many open source mutation identification software, SAMtools [9], SNVmix [16], and SOAPsnp [17] are a few widely used ones. SeqGene's mutation detection is implemented in a similar fashion to the pileup function in SAMtools but with a number of new filtering options. To identify SNPs, SeqGene's '*pileup*' function reads the alignment results in '.sam' format and reports chromosomal positions for candidate SNPs and indels. From the '*pileup*' output, SeqGene's '*snp*' function will filter the SNPs and indels based on a number of criteria: 1) the total coverage, i.e., the number of reads covering a candidate SNP (default 20); 2) the base quality, when the quality string is present, any base calling with low Phred quality will be removed from the coverage (default 10); 3) minor sequenced strand frequency, i.e., the proportion of reads covering both strands must reach certain threshold (default 0.1); and 4) mutated bases frequency, i.e., the proportion of mutated reads must be significant among all reads covering the position (default 0.25).

The multi-criteria SeqGene mutation filter is designed to be versatile to handle various exome-Seq and RNA-Seq projects. For example, in detecting somatic mutation in cancer samples, one can use a lower allele percentage threshold to account for altered ploidy of cancer samples. In high-depth targeted sequencing, one can increase the coverage threshold to improve the false discovery rate. In addition, since the SNP filter works with '.sam' file, it can work with sequencing data from many sequencing platforms and with various alignment software including Bowtie[18], BWA [19], and Novoalign [20].

### Mutation annotation and genotyping

The '*snp*' function also performs mutation annotation such as gene model annotation (upstream, downstream, UTRs, exon, intron, splice sites, etc), miRNA and other non-coding RNA annotation, consequence of the mutation (synonymous, non-synonymous, frame shift, non-sense etc), dbSNP annotation [21], hetero- or homo- zygosity and ASE on the mutation site. The ASE field lists the number of reads for each allele at all mutation positions. Using RNA-Seq data, users may use the ASE information to detect biased expressed variant alleles on heterozygous coding regions. For human samples, allele frequencies from 1000 Genome [15] data can be added into the annotation as well.

The '*genotyping*' function generates genotyping calls on the mutation positions across one or more samples. All positions that pass the SNP filter will be called either 'heterozygous mutations' or 'homozygous mutations'; positions that fail to pass the SNP filter will be labelled 'quality control' for unknown genotypes; positions that are not mutated is called either 'homozygous reference' or 'quality control' depending on whether the coverage is above or below the SNP filter threshold. One can use the '*genotyping*' function to aggregate mutations across multiple samples to identify mutations that match specific contrasts.

### Coverage (sequencing depth) quantification and visualization

The '*sam2wig*' function efficiently converts the alignment file into the per-base-resolution coverage file in '.wig' format. For exome-Seq, the '*exon_qc*' function report all the missing and defective regions with poor coverage, the quantile of the average exon coverage across exome (coverage sensitivity) and the percentage of total mapped reads aligned onto target exon regions (coverage specificity). For RNA-Seq, the '*rpkm*' function output the number of reads covering the genes and estimate the expression abundance using the average coverage as well as RPKM (reads per kilobase of exon model per million mapped reads) [8] as the normalized expression estimation for each transcript and exon. The '*phenotyping*' function aggregates one or more samples and generates the expression table for all transcripts and exons across all samples. The visualization of coverage and SNPs for each gene can be generated using the '*snpview*' function in scalable vector graphics (SVG) format which supports user interactions such as zooming and linking to Ensembl Genome Browser [22].

### eQTL

A quantitative trait locus (QTL) is a region of DNA that is associated with a particular phenotypic trait. eQTLs are genomic loci that regulate expression levels of mRNAs or proteins. By assaying gene expression and genetic variation simultaneously on a genome-wide basis in a large number of individuals, eQTL analysis can map the genetic factors that underpin individual differences in genome-wide gene expression pattern. Detecting eQTLs through RNA-Seq has been demonstrated as a robust and statistically powerful method in recent studies [23-25]. One of the most important applications of eQTL is to combine eQTL detection and genome-wide association (GWA) to identify specific genetic markers that are simultaneously associated with disease and eQTLs, as demonstrated in recent studies in asthma [26,27] and reviewed by Cookson et al. [28]. The '*eqtl*' function in SeqGene was computed on expression and genotype data using the '*lm*' function in the R '*stats*' package. The genotype data can be provided by the users or generated from the RNA-Seq data using the '*genotyping*' function. In the latter case, the genotyping are limited to those moderately or highly expressed genes on which a sufficient number of reads were mapped for reliable genotyping calls.

### Differentially expressed genes (DEGs)

A common application of RNA-Seq is to identify DEGs between two or more treatment groups. The '*diffexp*' function in SeqGene can compute fold change, Student's t-test p-value, Wilcoxon test p-value and false discovery rate (FDR) for all transcripts and exons between two treatment groups. For more complex study designs, one can directly work with the expression table generated using the '*phenotyping*' function and the methods borrowed from the microarray gene expression analysis, such as from Bioconductor's *limma *[29] package, to perform multiple group comparison on RNA-Seq data.

### Genotype-controlled differentially expressed genes (GCDEGs)

A more general way to describe a study design for identifying DEGs is a linear regression model, which describes the linear relationship between treatment variable *tr *and gene expression variable *e*. For each gene, the linear model is denoted as:(1)

where *n *is the number of samples, *e_i _*is the gene expression value of sample *i*, *tr_i _*is the treatment group of sample *i *(for example, it could be 'treated' or 'control'), *β*_0 _is the intercept parameter, *β *is slope parameter, and *ε_i _*is the error term for sample *i*. This linear model can describe multiple group comparison as well. The test for *β*≠0 is equivalent to a two-group Student's t-test (if assuming equal variance between the two groups for the Student's t test).

As shown in eQTL studies [23-25], the genotype differences among individuals could significantly impact the overall expression variation. The strong association between genotype and expression, however, could confound and obscure the treatment effect which is the main interests in DEGs. To address this problem, we proposed a new method incorporating genotypes as confounding variables to control for their effects in identifying DEGs in different treatment groups. Suppose a gene harbours *m *SNPs with its region, the so-called GCDEG is illustrated in a linear regression model as below:(2)

Where *m *is the number of SNPs within the gene region, *SNP_ij _*is the genotype of the *j*th SNP for the *i*th sample, *β_j _*is slope parameter for the *j*th SNP, *β*' is slope parameter for treatment after controlling for genotypes. Here we consider only the SNPs in gene regions. The GCDEG strategy is to test both parameter *β*≠0 in equation (1) and adjusted parameter *β'*≠0 in equation (2) and require both tests to be significant. The genotype information can be obtained by other sources. In fact, RNA-Seq data can be used for genotyping moderately to highly expressed genes. In SeqGene, the GCDEG method is implemented in the '*diffexp*' function which employs the linear mixed-effects model '*lme*' in R package '*nlme*'.

### Copy number variation

We implemented an interface in SeqGene to the '*DNAcopy*' package in Bioconductor [30] for CNV detection from exome-Seq data. In the '*cnv*' function, the log2 RPKM estimation of each exon was used as normalized probe signals for chromosomal segmentation and copy number calls. Note that intergenic and intronic CNV calls might not be accurate since these regions are not generally covered by the exome-Seq data. Also note that a reference (such as a normal DNA sample or the average of a group of pooled samples) is needed for absolute copy number calls.

### Pathway-based SNP-DEG association

Detecting significant SNP-expression association using eQTL is effective, however, it requires a large sample size (dozens and above) to generate sufficient statistical power for the genome-wide test. We therefore devised a new pathway topology-based strategy that is especially suited for DEG studies with limited sample size. The assumption of this method is that a SNP-harbouring gene (gSNP) may alter the regulation of the expression of itself and/or a downstream gene (gDEG). The significance of the SNP-DEG association is determined by the topological distance between a gSNP and a gDEG in a regulatory pathway. Therefore a cis-acting SNP (i.e., gSNP and gDEG is the same gene) is considered most significant. The further down the pathway, the less significant of the association. To calculate the distance between any gSNP and gDEG pair, we merge all KEGG pathways [31] graphs into a single directed graph *G *which contains *N *genes (nodes). Using Johnson's algorithm [32], we compute the distance matrix *d *for each pair of genes, where *d_i, j _*is the shortest distance from gene *i *to gene *j*. If there is no path from gene *i *to gene *j*, *d_i, j _*is set to equal to *N*. The shortest distance from gSNP to gDEG is notated as *d_gSNP, gDEG_*, which is used as the test statistic for the SNP-DEG association using distance matrix *d *as the background. The p-value for *d_gSNP, gDEG _*is defined by:(3)

where *I*(*x*) is the indicator function .

## Implementation

SeqGene's major functions (Figure 1) were implemented in Python. Some functions such as CNV, DEG, GCDEG, eQTL and KEGG pathway also require R and some Bioconductor packages to process their statistical components and graph theory algorithms. The source code is modularly and loosely structured of those components, and therefore, it is relative easy to add new functions to the package. SeqGene supports a simple command-line interface and can also be run in a customized batch processing mode. SeqGene is independent of any specific alignment software; one may choose to use any alignment software as long as the alignment output is in the cross-platform SAM (Sequence Alignment/Map) format [9]. This alignment-independent design allowed SeqGene to support both paired-end reads and single reads generated from most high-throughput sequencing platforms.

SeqGene's algorithms were optimized and one can expect the analysis tasks finish within minutes to a few hours. For example, SeqGene's SNP pileup function runs at similar speed as SAMtools [9] which was implemented in C. The memory fingerprint of SeqGene is well-controlled such that a workstation with 16 G RAM is sufficient for most projects. On multi-processor workstations, one can run multiple jobs of SeqGene to achieve parallel speedup. The annotation packages for latest Ensembl Human, Mouse and Rat [22], and UCSC Genome Browser hg18 and hg19 [33], were pre-built and can be downloaded from the project website. In addition, SeqGene has a function '*buildannot*' and corresponding instructions for building additional annotation packages for other organisms from Ensembl, UCSC Genome Browser or arbitrary assemblies.

## Results

### Trio-family exome sequencing showed robust SNP identification and genotyping using SeqGene

To test SeqGene's mutation detection algorithm, we performed exome-Seq on a trio family (father, mother, and daughter) with no history of inherited diseases. Genomic DNA was extracted from saliva using Oragene DNA Kit (DNAgenotek Inc., Ontario, Canada) and sonicated using bioruptor (Diagenode Inc., Denville, NJ). Sonicated DNA (3 ug) was used to make a library for paired-end sequencing (Illumina Inc., San Diego, CA) and fragments with approximately 200 -250 bp insert DNA were select and amplified. After quality control, 750 ng of the library was hybridized to biotinylated cRNA oligonucleotide baits from the SureSelect Human All Exon kit (Agilent Technologies Inc., Santa Clara, CA), purified by streptavidin-bound magnetic beads, and amplified for 12 cycles. After purification, the library was paired-end (80 × 80 bp) sequenced using Illumina Genome Analyzer IIx (Illumina Inc., San Diego, CA). The exome probes cover 38 Mb of human genome corresponding to the exons and flanking intronic regions of 23,739 genes in the National Center for Biotechnology Information Consensus CDS database (September 2009 release) and also cover 700 miRNAs from the Sanger v13 database and 300 noncoding RNAs from Ensembl GRCh37.56.

The sequencing reads were aligned to Human reference genome (Ensembl GRCh37.56) using Novoalign [20] with default alignment parameters. Mutation identification was performed using SeqGene, SAMtools [9], and VarScan [34] respectively. We used a family-wise SNP filter which ignores any mutations that failed genotyping due to quality control on any of the family members. Table 1 showed the parameters that we used in SeqGene, SAMtools, and VarScan for mutation filters.

**Table 1:** SNP and indel identification parameters for VarScan, SAMtools and SeqGene in the trio family analysis

	VarScan	SAMtools	SeqGene
SNP pileup	SAMtools pileup (default, mapping quality > 10)	SAMtools pileup (default, mapping quality > 10)	SeqGene pileup (default)

SNP filter	Coverage: > 20, 10 Average quality: > 20 Mutated bases frequency: > 25% p-value: < 1E-6	Default filter (SAMtools varfilter) Coverage: > 20, 10 SNP quality > 20	Coverage: > 20, 10 Bases Phred quality: > 10 Mutated bases frequency: > 25% Minor sequenced strand: > 10%

Family-wise filter	Ignore positions with at least one 'quality control' across the family	Ignore positions with at least one 'quality control' across the family	Ignore positions with at least one 'quality control' across the family

**Figure 2:**
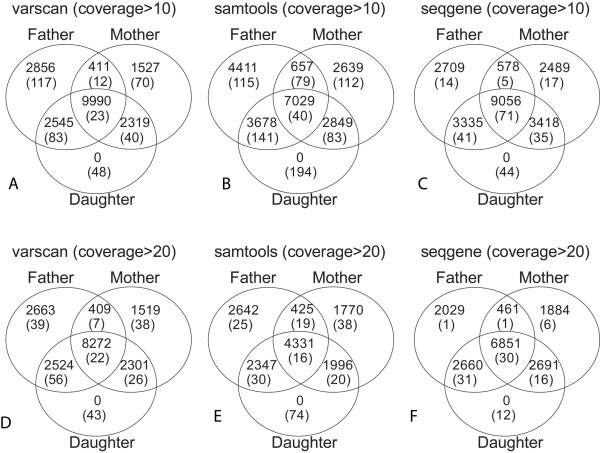
**Distribution of SNP positions across the trio using VarScan, SAMtools and SeqGene**. In the Venn diagrams, the numbers shown in the overlap indicate shared mutations between the family members. Numbers not in parentheses are the number of SNP positions that passed genotype pedigree check; numbers in parentheses are the number of SNPs positions that failed genotype pedigree check, i.e., Mendelian errors.

Mendelian error rates of the identified SNPs were calculated as an indirect indication of genotyping quality. As demonstrated in Figure 2 and Table 2, SeqGene's mutation identification algorithm had significant lower Mendelian error rates while maintaining similar mutation discovery power comparing with SAMtools. We compared the number of SNPs (after family-wise filter) between VarScan, SAMtools and SeqGene using coverage > 10 and coverage > 20 for the three samples, and found that the number of SNPs that passed pedigree check by SeqGene are considerably higher than those by SAMtools for all cases, expect one (Father sample, coverage > 10) where SeqGene identified slightly lower number of SNPs. More importantly, the number of SNPs that failed pedigree check (Mendelian errors) was reduced by around 50% in SeqGene as compared to SAMtools. For example in Figure 2E, SAMtools identified 72 mutations in the daughter which were not found in any of her parents, whereas SeqGene identified only 12 such Mendelian errors (Figure 2F). SeqGene also compares favorably to VarScan as shown in Table 2 and Figure 2. With similar numbers of identified SNPs, the Mendelian error rates are consistently lower in SeqGene than in VarScan. In addition, the performance of VarScan is consistently better than that of SAMtools.

**Table 2:** Number of SNPs and Mendelian error rates using Varscan, SAMtools and SeqGene

		VarScan			SAMtools			SeqGene		
		**Father**	**Mother**	**Daughter**	**Father**	**Mother**	**Daughter**	**Father**	**Mother**	**Daughter**

	**After SNP Filter**	26458	19814	20788	39657	24384	26971	23522	22776	24097

**Coverage** **> 10**	**After Family Filter**	16037 (235)	14392 (145)	15048 (194)	15775 (375)	13174 (324)	13556 (458)	15678 (131)	15541 (128)	15809 (191)

	**Mendelian Error Rate (%)**	**1.5**	**1.0**	**1.3**	**2.3**	**2.4**	**3.6**	**0.8**	**0.8**	**1.2**

	**After SNP Filter**	23444	16317	17639	27814	15797	18060	18805	16354	17889

**Coverage** **> 20**	**After Family Filter**	13992 (124)	12594 (93)	13244 (147)	9745 (90)	8522 (93)	8674 (140)	12001 (63)	11887 (53)	12202 (89)

	**Mendelian Error Rate (%)**	**0.9**	**0.7**	**1.1**	**0.9**	**1.1**	**1.6**	**0.5**	**0.4**	**0.7**

**Figure 3:**
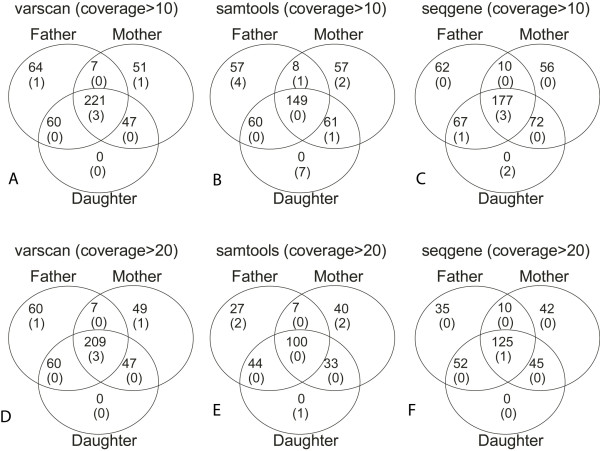
**Distribution of short indels across the trio using VarScan, SAMtools and SeqGene**. In the Venn diagrams, the numbers shown in the overlap indicate shared mutations between the family members. Numbers not in parentheses are the number of indels that passed genotype pedigree check; numbers in parentheses are the number of indels that failed genotype pedigree check, i.e., Mendelian errors.

Using the same settings in Table 1, we generated the list of indels using VarScan, SAMtools, and SeqGene respectively and we compared their performance. SeqGene and VarScan consistently outperform SAMtools in terms of Medelian error rates and the number of indels detected as shown in Table 3 and Figure 3. Mixed results were observed when comparing SeqGene and VarScan for indel filtering. Under coverage > 10, SeqGene generates slightly higher error rate than VarScan. Under coverage > 20, SeqGene generates lower number of indels than VarScan. However the error rates (0.4%) of SeqGene are also lower than those from VarScan (0.9%-1.2%).

**Table 3:** Number of indels and Mendelian error rates using Varscan, SAMtools and SeqGene

		VarScan			SAMtools			SeqGene		
		**Father**	**Mother**	**Daughter**	**Father**	**Mother**	**Daughter**	**Father**	**Mother**	**Daughter**

	**After SNP Filter**	696	522	522	539	560	634	637	628	703

**Coverage** **> 10**	**After Family Filter**	356 (4)	330 (4)	331 (3)	279 (5)	279 (4)	278 (8)	320 (4)	318 (3)	322 (6)

	**Mendelian Error Rate (%)**	**1.1**	**1.2**	**0.9**	**1.8**	**1.4**	**2.9**	**1.2**	**0.9**	**1.9**

	**After SNP Filter**	690	503	506	389	355	413	478	416	472

**Coverage** **> 20**	**After Family Filter**	340 (4)	316 (4)	319 (3)	180 (2)	182 (2)	178 (1)	223 (1)	223 (1)	223 (1)

	**Mendelian Error Rate (%)**	**1.2**	**1.3**	**0.9**	**1.1**	**1.1**	**0.6**	**0.4**	**0.4**	**0.4**

In addition, SeqGene's '*snp*' function can provide detailed annotations to the SNPs based on the gene model categorization (such as 5' UTR, missense, nonsense, intron, splice site, 3' UTR, intergenic, frameshift, synonymous). The resultant annotation file can be aggregated into cross-sample format using 'genotyping' function. Other filtering and analysis with the annotation files are possible. For example, the users can obtain non-synonymous mutations using 'polyphen' function and the output can be submitted to PolyPhen server [35] for further processing.

### Identify eQTLs in HapMap RNA-Seq data

In this example, we showed the SeqGene's capability on expression quantification and eQTL by reanalyzing a public data set from the international HapMap project [23,36]. The data set contains the RNA-Seq samples of 60 CEU individuals (HapMap individuals of European descent). The mRNA fraction of the transcriptome of lymphoblastoid cell lines (LCLs) from those samples were sequenced using 37-base pairs (bp) paired-end Illumina sequencing. Each individual's transcriptome was sequenced in one lane of an Illumina GAII analyzer.

**Figure 4:**
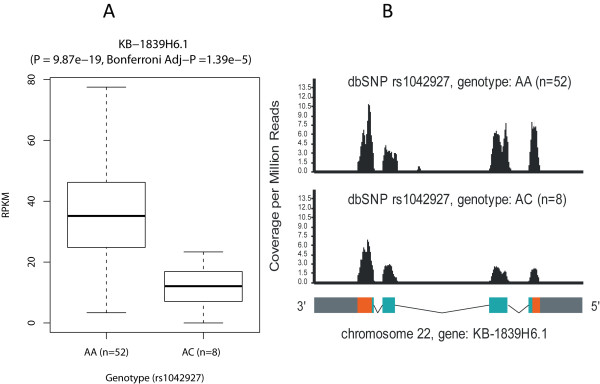
**An example of significant eQTL identified using SeqGene**. The HapMap individuals were stratified based on their genotypes at *rs1042927 *(dbSNP entry nubmer). We found 52 individuals are of genotype 'AA' and the other eight samples are of genotype 'AC'. We compared the expression levels of the gene *KB-1839H6.1 *in the two groups. (A). The *KB-1839H6.1 *gene expression level (RPKM) in the two groups (with 'AA' or 'AC' genotype at *rs1042927*). The expression levels were quantified using SeqGene's '*rpkm*' function. (B). The aggregated coverage in the two groups illustrated on the gene model. The coverage is normalized to 'Coverage per Million Reads'. This plot was generated using the SeqGene's '*snpview*' function. Gene models for every transcript were displayed at the bottom as flanking regions (gray), UTRs (orange), CDS (green) and introns (lines).

We aligned the short reads to the UCSC Genome Browser hg19 human reference genome [33] using Tophat [37], which can automatically detect and align the short reads to candidate exon-exon junctions. We use SeqGene's '*sam2wig*' and '*rpkm*' functions to quantify gene expression of individual samples. SeqGene's '*phenotyping*' function is then used to tabulate gene expression across multiple RNA-Seq samples. The genotype information was obtained from the international HapMap project [36]. The expression profiles from multiple samples, along with the genotypes, were processed using SeqGene's '*eqtl*' function, which is capable to report both cis- (locally) and trans- (at a distance) eQTLs to a gene. Figure 4 showed an example of a strong eQTL that affects the expression level of gene *KB-1839H6.1*. The genetic marker is dbSNP entry *rs1042927*, which is located on chromosome 11, whereas the gene *KB-1839H6.1 *is located on chromosome 22. Therefore, this is a trans-eQTL which maps far from the location of its gene-of-origin gene. The Bonferroni-adjusted p-value of this eQTL is 1.39e-5. The '*snpview*' function in SeqGene will further display the wiggle plot superimposed on the gene model, as shown in Figure 4.

### Identify GCDEGs from public RNA-Seq dataset

**Figure 5:**
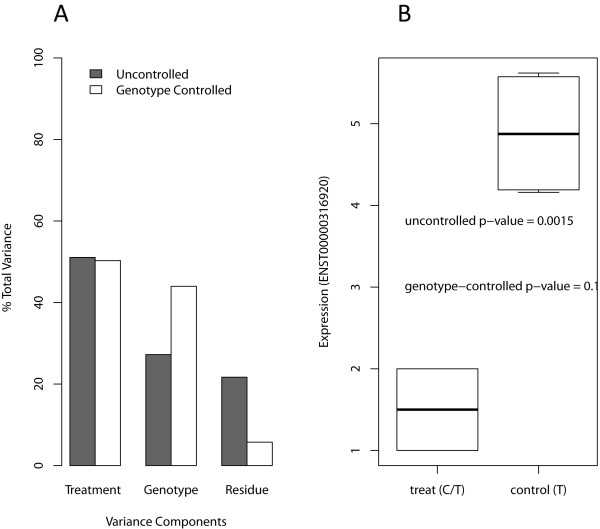
**Identifying GCDEGs using SeqGene on RNA-Seq data**. (A). Comparing variance components of GCDEGs (white bars) with DEGs (dark bars). The variance components were computed for three factors (Treatment, Genotype, and Residue) using SeqGene with the '*lme*' function in R package '*nlme*' and the '*varcomp*' function in R package '*ape*'. In this example, the uncontrolled DEGs were detected using Student's t-test p < 0.01. The controlled GCDEGs satisfied both Student's t-test p < 0.01 and the adjusted p-value < 0.01. We observed that Residue variance is significantly reduced in GCDEGs. (B). An example gene showing the effect of avoiding the confounding genotype factor. In this example, the treatment and genotype are completely confounded in that the treatment samples had genotype (C/T) and the control samples had genotype (T/T). The uncontrolled p-value is 0.0015, whereas it is no longer significant after genotype controlling.

We demonstrate the novel GCDEG method in SeqGene by reanalyzing a recently published RNA-Seq dataset [38]. The samples contain double poly(A)-selected RNA from primary CD4+ T cells with both activated and untreated conditions. We aligned the short reads to the UCSC Genome Browser hg19 human reference sequences [33] using Tophat [37]. The genome-wide gene expression profiling were performed using '*sam2wig*', '*rpkm*', and '*phenotyping*' functions. Then the '*diffexp*' function was used to perform two-group comparison between the 'stimulated' and 'unstimulated' samples to identify DEGs and GCDEGs. DEGs were selected using Student's t-test p-value < 0.01. GCDEGs were selected using two cutoff values: Student's t-test p-value < 0.01 and genotype-controlled p-value < 0.01. We compared the variance components on the selected DEGs and GCDEGs using SeqGene by the'*varcomp*' function in R package '*ape*'. Figure 5A showed the variance components for DEGs and GCDEGs for the 'treatment', 'genotype' and 'residue' components, respectively. We observed significant residual error reduction in the GCDEGs method as compared to DEGs, and more variance was explained by the 'genotype' component in the GCDEGs. Figure 5B showed an example gene in which the treatment effect is badly confounded with genotype. This example illustrated that GCDEGs can help reduce errors and avoid DEGs that are confounded with genotype.

### Identify somatic mutation and copy number variation from Acute Myeloid Leukemia (AML) exome sequencing data

We reanalysed the exome sequencing data from a recent study by Yan et al. [39]. The dataset contains nine paired samples of AML-M5 cases with bone marrow cancer samples obtained at the time of diagnosis and control peripheral blood specimens obtained after complete remission. Five additional AML-M5 cases without matched normal samples were also analyzed. The captured target in each exome was 24 Mb. From EBI sequence Read Archive with submission ID SRP005624, we downloaded a total of 96 lane of sequencing runs in. fastq format and aligned the reads to Human hg19 reference assembly using bwa [19]. Table 4 shows the alignment coverage report using 'exon_qc' function for the nine bone marrow samples and their corresponding blood samples. The average coverage for the samples is in the range 44 fold to 117 fold on refseq exons. 61% to 68% of exons in refseq were covered at > 10 fold on average. 65% to 70% of exons in refseq were covered with > 5 fold on average. We next carried out mutation detection and filtering using seqgene's 'sam2wig', 'sam2pileup', 'snp' and 'genotyping' functions to obtain the genotype in a tabulated format across 23 samples. We obtained rare somatic mutations in bone marrow sample by filtering dbSNP 131 and the germline mutations in blood sample. Table 5 lists three rare somatic missense mutations for DNA methyltransferase gene DNMT3A which is consistent with the original report. Note that one mutation may be located at multiple transcripts and therefore was annotated multiple times.

**Table 4:** Quality control of AML samples annotated on refseq, Human hg19

	Blood1	Blood2	Blood3	Blood4	Blood5	Blood6	Blood7	Blood8	Blood9
mde	48	47	50	47	87	86	99	102	95

ec5 (%)	65	68	67	69	69	69	69	69	69

ec10(%)	61	65	65	66	66	66	67	67	67

	**Bone1**	**Bone2**	**Bone3**	**Bone4**	**Bone5**	**Bone6**	**Bone7**	**Bone8**	**Bone9**

mde	46	45	44	44	79	86	75	77	117

ec5 (%)	68	68	68	69	69	69	68	68	70

ec10(%)	65	65	65	65	67	66	66	65	68

mde: mean depth on exons; ed5: percentage of exons with at average depth greater than 5; ed10: percentage of exons with at average depth greater than 10;

**Table 5:** Three novel missense somatic mutations of DNMT3A identified in 23 samples using seqgene (cov > 10)

transcript	position (hg19)	position transcript	codon number	amino acid change	ref	bl3	bm3	bl9	bm9	ex5
NM_022552	chr2:25457197	2947	897	Val- > Asp	A	A	A/T			

NM_175629	chr2:25457197	3028	897	Val- > Asp	A	A	A/T			

NM_153759	chr2:25457197	2237	708	Val- > Asp	A	A	A/T			

NM_022552	chr2:25467449	1884	543	Gly- > Cys	C					A/C

NM_175629	chr2:25467449	1965	543	Gly- > Cys	C					A/C

NM_153759	chr2:25467449	1174	354	Gly- > Cys	C					A/C

NM_022552	chr2:25457242	2902	882	Arg- > His	C			C/T	C	

NM_175629	chr2:25457242	2983	882	Arg- > His	C			C/T	C	

NM_153759	chr2:25457242	2192	693	Arg- > His	C			C/T	C	

**Figure 6:**
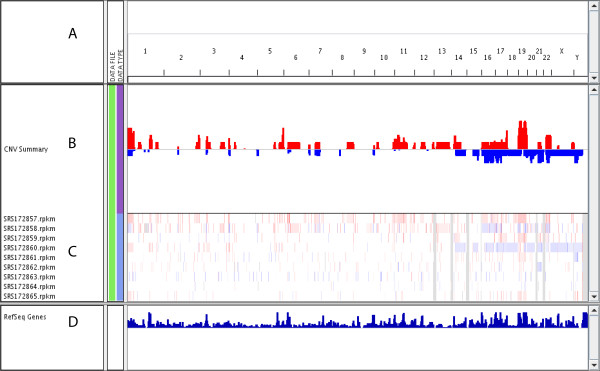
**IGV snapshot shows CNV identified using SeqGene CNV function on 9 pair of AML exome-Seq data**. (A). Coordinates of Human hg19 assembly displayed. (B). Copy number aggregated across 9 pair of samples, genomic amplification is displayed in red bars and genomic deletion is displayed in blue bars; the height of color bars indicate the number of samples that displayed genomic aberrations. (C). Heatmap shows the predicted genomic segments (colored regions) and breakpoints using seqgene's cnv function; The colors indicate and mean marker signals with blue represents negative values and red represents positive values; (D) Density of refseq genes across genome.

We then performed CNV analysis on the nine pairs of samples using the 'cnv' function. For each cancer sample, its control blood sample was used to normalize the signals. The 'cnv' function generated results in '.seg' format which include genomic break points estimation and mean signals for all genomic regions. The output '.seg' file was loaded in to Integrative Genomics Viewer (IGV) [40] for visualization and the results were shown in Figure 6. From the copy number aggregation view (Figure 6A), we observed recurrent (more than 2 cases) copy number gain on chromosome 5q, 17q25, 19, and 22. Particularly, four out of the nine samples show amplification on significant portions of chromosome 19. This results indicate chromosome 19 amplifications may be a hallmark of AML as reported in an earlier study by Nimer et al. [41]. It should be noted that exome-Seq experiments focus only on exons and generate very uneven coverage across exons due partially to sequence capture biases. However, analysis using exome-Seq data may still shed light on copy number variations beyond the exons when paired control samples are available and a reasonable breakpoint estimation algorithm such as Circular Binary Segmentation (CBS) [30] is used.

We also recorded run time and memory usage when performing different tasks for this relatively large scale project (Table 6). Note that currently all tasks can finish in reasonable time except for global trans-eQTL calculation which needs days of calculation using on multiple CPUs.

**Table 6:** Computing performance of major functions using 23 exome sequencing samples on a 16 CPU workstation

Script	Function	#CPU	Peak RAM (Gb)	Time	Notes
**exon_qc**	quality control	1	6	10-20 min	Per sample

**sam2wig**	generate wig file	1	2	20-50 min	Per sample

**sam2pileup**	generate pileup file	1	3-7	1-4 hours	Per sample

**snp**	annotate and filter snp and indels	1	4	2-8 min	Per sample

**rpkm**	quantify coverage on gene model	1	4	4-16 min	Per sample

**cnv**	copy number variation	1	4	5 min	Per sample

**genotyping**	genotyping file across samples	1	8	4 hour	Across 23 samples

**phenotyping**	coverage (expression) across samples	1	5	10 min	Across 23 samples

**eqtl -m cis**	Cis- EQTL	1	3	1 hour	1000 genome data

**eqtl -m trans**	Trans-EQTL	16 *	3	7 days	1000 genome data

### Comparing mutations discovered using paired exome-Seq and RNA-Seq samples

We reanalyzed paired RNA-Seq and exome-Seq data derived from breast cancer cell line, HCC1954 reported by Zhao et al. [42]. Our goal of this integrated analysis is to estimate the correlation between the mutations identified using paired DNA and RNA sequencing of cancer samples. Exome-Seq was performed on Roche 454 platform and RNA-Seq was performed on Illumina GAII platform. The datasets were downloaded from the EBI Sequence Read Archive (ERA) with submission ID ERA010917 for exome-Seq data and ERA011762 for RNA-Seq data.

We applied Tophat [37] for the RNA-Seq spliced alignment and bwa-sw [19] for exome-Seq long reads alignment on Human hg19 assembly. We generated quality control reports using 'exon_qc' function on the two samples respectively. The exome-Seq alignment shows that 59.6% of the refseq exons were covered at ≥ 5 fold and the mean coverage on all exons is 24 fold; RNA-Seq yields 4.8% of the refseq exons with ≥ 5 fold coverage and the mean coverage on all exons is only 1.9 fold. Quality control of the RNA sample shows that 3.4% of the 48 million aligned reads were located on intergenic regions or introns, indicating possible contamination of the RNA sample. We then performed mutation discovery on the paired samples using SeqGene. After applying quality control filtering to obtain SNPs that passed quality control in both exome and RNA samples, we identified 29 the SNPs on coding regions and UTRs. We then compared the genotypes of the 29 SNPs between exome and RNA for genotyping consistency. The results were summarized in Table 7. The total number of matched mutations between exome and RNA samples is 20 out of the 29 SNPs. Five heterozygous SNPs (called from DNA) showing homozygous expression pattern from RNA are ASE candidates. Three homozygous SNPs (called from DNA) showing heterozygous expression on RNA sample are obvious genotyping inconsistencies. The lower than expected number of SNPs and low genotyping consistency between exome and RNA genotyping may be due to a number of factors such as biased exome sequence capture, possible contamination of RNA sample, misplaced alignment, and sequencing errors.

**Table 7:** Number of SNPs between paired RNA-Seq and Exome-Seq samples

	RNA-hom	RNA-het
DNA-hom	17 (0)^c^	3^a^

DNA-het	5^b^	3 (1)^c^

a:genotyping inconsistency between DNA and RNA, b: candidate ASE, c: number of inconsistent genotypes

## Conclusion

We developed an open-source software tool, SeqGene, to support massively parallel exome-Seq and RNA-Seq data analysis. SeqGene supports functions of base-resolution read coverage, quality control, SNP/indel identification and annotation, RNA and DNA depth quantification, ASE, CNV, eQTL, DEG, and KEGG pathway analysis. Among the many functions of SeqGene, we have also implemented novel methods for genotype-controlled differentially expressed genes (GCDEGs) identification, and SNP-DEG association test using KEGG pathways. We have demonstrated that SeqGene is a useful data mining tool to support a wide variety of analysis tasks in exome-Seq and RNA-Seq data.

## Abbreviations

AML: acute myeloid leukemia; ASE: allele specific expression; CBS: circular binary segmentation; CNV: copy number variation; DEG: differentially expressed gene; eQTL: expression quantitative trait locus; FDR: false discovery rate; GCDEG: genotype-controlled differentially expressed gene; GWA: genome-wide association; IGV: integrative genomics viewer; LCL: lymphoblastoid cell lines; miRNA: MicroRNA; QTL: quantitative trait locus; RPKM: reads per kilobase of exon model per million mapped reads; SNP: single nucleotide polymorphism; SVG: scalable vector graphics; UTR: untranslated regions.

## Competing interests

The authors declare that they have no competing interests.

## Availability and requirements

The SeqGene software, annotation packages and user's manual can be accessed at http://seqgene.sourceforge.net. SeqGene requires Python 2.6 or 2.7 and CNV, DEG, GCDEG, eQTL and KEGG pathway functions also require R and certain Bioconductor packages. SeqGene is cross-platform software and has been tested on Linux-, Macintosh- and Windows- based workstations. SeqGene is free for academic use and require a license from the author for commercial applications.

## Authors' contributions

XD designed and implemented the SeqGene, performed the analysis and wrote the manuscript.
